# Calixresorcin[4]arene-Mediated Transport of Pb(II) Ions through Polymer Inclusion Membrane

**DOI:** 10.3390/membranes11040285

**Published:** 2021-04-13

**Authors:** Joanna Konczyk, Wojciech Ciesielski

**Affiliations:** Faculty of Science and Technology, Jan Dlugosz University in Czestochowa, 13/15 Armii Krajowej Str., 42-200 Czestochowa, Poland; wc@ujd.edu.pl

**Keywords:** polymer inclusion membrane, calixresorcin[4]arene, lead, heavy metal

## Abstract

A facilitated transport of Pb(II) through polymer inclusion membrane (PIM) containing 1,8,15,22-tetra(1-heptyl)-calixresorcin[4]arene and its tetra- and octasubstituted derivatives containing phosphoryl, thiophosphoryl or ester groups as an ion carrier was investigated. The efficiency of Pb(II) removal from aqueous nitrate solutions was considered as a function of the composition of membrane (effect of polymer, plasticizer, and carrier), feed (effect of initial metal concentration and presence of other metal ions) and stripping phases, and temperature of the process conducting. Two kinetic models were applied for the transport description. The highest Pb(II) ions removal efficiency was obtained for the membrane with tetrathiophosphorylated heptyl-calixresorcin[4]arene as an ion carrier. The activation energy value, found from Eyring plot to be equal 38.7 ± 1.3 kJ/mol, suggests that the transport process is controllable both by diffusion and chemical reaction. The competitive transport of Pb(II) over Zn(II), Cd(II), and Cr(III) ions across PIMs under the optimal conditions was also performed. It was found that the Cr(III) ions’ presence in the feed phase disturb effective re-extraction of Pb(II) ions from membrane to stripping phase. Better stability of PIM-type than SLM-type membrane was found.

## 1. Introduction

Global economic development, which brings new technologies and materials improving the quality of human life, leads to adverse changes in the natural environment. Despite the fact that many countries implement adequate laws mandating reductions in the emission of pollutants to the environment, the presence of substances dangerous for organisms in the environment remains a valid problem. Heavy metals form the largest group of inorganic contaminants of surface and underground waters, and the concentration values of the most harmful ones, such as lead, cadmium, zinc and chromium in water are indicators of the water quality [1]. The presence of lead ions in environmental waters worldwide is the effect of mining and processing of fossil fuels that contain lead, production and utilization of products containing this element (e.g., dyestuffs, glass, batteries, water tanks, water distribution pipelines, electric cables, PV cells, infrared radiation detectors and artificial fertilizers), and inadequate waste storage and discharge of post-production wastewater. Exposing the body to higher daily doses of lead may cause severe intoxication, multi-organ disease, or in extreme cases—death [2].

Extraction processes based on liquid membranes have attracted much attention as separation methods that could replace the currently used technologies for environmental water, process solution and industrial wastewater treatment [3]. Liquid membranes are better than solvent-based extraction and solid membranes because they can be designed so that they are highly selective for a specific substance at a fairly low consumption of the extractant/carrier. Moreover, adequate transport conditions eliminate the need to divide the process into many stages, as happens in solvent extraction. Recent decades saw intensive research efforts focusing on polymer inclusion membranes (PIMs) in the aspect of their use for selective release of harmful substances from post-production waste flux, industrial wastewater or environmental waters. The literature data suggest that the membranes enable effective removal of a wide range of organic and inorganic substances, both neutral and ionic, including heavy metals, from synthetic and real solutions [4,5,6,7,8,9,10,11].

The effectiveness of the polymer inclusion membrane depends on its composition and morphology [6,7,12]. The selection of the right polymer, plasticizer, carrier and sometimes an addition of a modifier or a synergic medium greatly affects the membrane pertraction efficiency. Polymers play a key role in ensuring the membranes mechanical strength, and their characteristics determine the membrane permeability and durability. In the majority of studies on PIM, polymer supports are used, made of amorphous polyvinyl chloride (PCV) or highly crystalline cellulose triacetate (CTA). PCV has functional polar groups C–Cl that cause stronger interactions of non-specific dispersion forces than intermolecular interactions. In turn, CTA is a polar polymer with hydroxyl and acetyl groups capable of forming hydrogen bonds. The plasticizers role is to improve elasticity and mechanical strength of the polymer support through the penetration between the polymer molecules and reduction in the power of intermolecular forces, consequently increasing the distance between the polymer molecules. Although there are many plasticizers available on the market, only some of them were tested for their use in PIM. The most common ones include *o*-nitrophenyl octyl ether, *o*-nitrophenyl pentyl ether, dioctyl adipate, dioctyl phthalate and methyltrialkylammonium chloride (Aliquat 336). Chemical compounds highly soluble in a liquid membrane, non-soluble in aqueous solutions and with reversible interaction with the transported ingredient, can be the metal ion carriers in the transport through polymer inclusion membranes. Basically, the same organic ligands that are used as extractants in solvent extraction are also the ion carriers in PIMs. Besides the commercially available extractants/ carriers of metal ions, newly synthesized chemical compounds, including macrocyclic and macromolecular compounds far more selective towards many metal ions than traditional carriers, are used for the preparation of liquid membranes [6,7,13,14]. Modified calixresorcinarenes (also named resorcinarenes or resorcarenes), which are calixarene derivatives with resorcinol groups, revealed high affinity to heavy metal ions during extraction to the liquid or solid phase [15,16,17,18,19,20,21]. The abilities of resorcinarenes to complex with metal ions differ from most popular calixarenes, resulting most of all from differences in their molecule structure [22]. The transport of metal ions through liquid membranes containing resorcinarenes as ion carriers is not a thoroughly investigated process and is described only in a few papers [18,23,24,25]. Benosmane et al. [23] and Ugur et al. [18,24] studied the ability of non-modified resorcin[4]arenes or their derivatives modified through the macrocyclic ring bridge to release Zn(II), Cd(II) and Pb(II) ions from aqueous solutions. They demonstrated that resorcin[4]arene, with a longer alkyl chain of a more hydrophobic nature, releases Pb(II) and Zn(II) ions more efficiently than a carrier with a shorter alkyl chain [23]. The substitution of the long alkyl by the ethereal alkyl [23] or phenyl groups [25] strengthened the affinity to Pb(II) ions and improved the effectiveness of the ions flux through PIMs, as compared to resorcinarenes with a straight alkyl chain. Moreover, Benosmane et al. [26] demonstrated a significant increase in the thermal stability of PIM following an addition of resorcin[4]arene to the polymer support.

The purpose of this paper was to evaluate the possibilities of using calixresorcin[4]arenes modified not by the methylene bridge but by resorcinol hydroxyl groups with four or eight phosphoryl, thiophosphoryl or ester groups in the role of Pb(II) ion carriers through polymer inclusion membranes and determination of the influence of basic process parameters on the transport efficiency. The obtained results will help to answer the question of whether the tested membranes can be used to effectively remove selected heavy metal ions from synthetic aqueous solutions and thus provide a background for the development of a technology for the purification of real solutions.

## 2. Materials and Methods

### 2.1. Reagents and Apparatus

**Figure 1:**
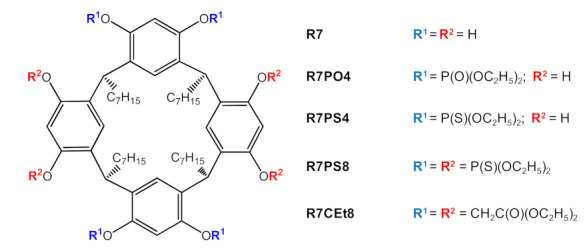
Chemical structure of the calixresorcin[4]arene carriers.

Deionized water, with medium electrolytic conductivity not exceeding 1 µS/cm at 20 °C, was used for preparing aqueous solutions, along with the following compounds of analytical grade: Pb(NO_3_)_2_ (POCh, Gliwice, Poland), Zn(NO_3_)_2_ 6H_2_O (POCh), Cd(NO_3_)_2_ 4H_2_O (Acros Organics, Geel, Belgium), Cr(NO_3_)_3_ 9H_2_O (Acros), HNO_3_ (POCh), HCl (Chempur, Piekary Slaskie, Poland), H_2_SO_4_ (POCh), CH_3_COOH (POCh), CH_3_COONa (Chempur), EDTA-Na (Sigma-Aldrich, Saint Louis, MO, USA) and 2-[4-(2-hydroxyethyl)-1- piperazinyl]-etanosulfonic acid (HEPES, Sigma-Aldrich). The following compounds were used for preparing the membranes: analytical grade trichloromethane (POCh) as the carrier solvent, *o*-nitrophenyl octyl ether (o-NPOE) and *o*-nitrophenyl pentyl ether (o-NPPE) with ≥99% purity (Fluka, Busch, Switzerland), bis(2-ethylhexyl) adipate (DOA) with 99% purity (Sigma-Aldrich) as plasticizers, cellulose triacetate (CTA, Fluka) as the polymer support and calixresorcin[4]renes with the structures shown in Figure 1. Synthesis of the carriers was described earlier in the papers [16,17].

Immobilized supported liquid membranes (SLM) were prepared from 25 µm thick Celgard 2500 type (Hoechst Celanese Corporation, Charlotte, NC, USA) of microfiltration polypropylene membrane sheets, with 45% porosity, pore curvature of 2.22 and pore diameter between 0.04 and 0.4 µm.

### 2.2. Membranes Preparation

Polymer inclusion membranes (PIM) were prepared by solvent evaporation from the solution developed by dissolving adequate quantities of the polymer, plasticizer and resorcin[4]arene in trichloromethane. The obtained mixture was poured onto a glass ring, with a 5 cm diameter, fixed to a flat plate with CTA solution in trichloromethane and left for 12 h until the solvent evaporated completely. The membrane created this way was left for the subsequent period of 12 h in a beaker with distiller water to smoothen the membrane surface. The thickness of the obtained membranes was measured in five points with an ultrameter (A 2002M type from Inco-Veritas, Warsaw, Poland). Supported liquid membranes (SLM) were prepared by placing a ring (5 cm diameter), cut out from Celgard 2500 sheet, for 12 h in the right carrier solution in trichloromethane.

### 2.3. Transport Studies

Metal ions were transported through the polymer inclusion membranes in a special system composed of two tanks, each holding 60 cm^3^ of the feed and the stripping phase, connected by a membrane with the effective area of 4.5 cm^2^, placed in a water bath ensuring the maintenance of the process constant temperature (25.0 ± 0.1 °C, except for the studies on the temperature influence on the transport kinetics, when the measurements were carried out at five different temperatures ranging from 25.0 to 50.0 ± 0.1 °C). The content of the tanks was mixed at a constant rate amounting to 600 rpms, while the pH of the feed solution was controlled by CX-505 pH-meter (Elmetron, Zabrze, Poland) with the ERH-12-6 electrode (Elmetron) and maintained at a constant level by adding small quantities of 0.10 M (M = mol/dm^3^) HNO_3_ or 0.10 M HEPES in water. At the right intervals, 0.5 or 1.0 cm^3^ volume samples were collected from the feed and the stripping phase, and they were subjected to a quantitative analysis for the content of metals, using atomic absorption spectrometry (Solaar 939 spectrometer, Unicam, Offenbach/Main, Germany). Two independent experiments were carried out on each stage of the test, using two separately synthesized membranes.

### 2.4. Membrane Characterization

The chemical composition of the membranes was analyzed with an X-Ray fluorescence sequence spectroscope with wave dispersion (WD-XRF) Rikagu ZSX Primus II (Rikagu, Wrocław, Poland). SEM imaging was performed with an electron scanning microscope Quanta 3D FEG with a field emission (FEI Company, Hillsboro, OR, USA). The observations of the analyzed samples were carried out following their coating with a 10 nm gold film in a high-vacuum (HV) mode at an accelerating voltage amounting to 20 kV, using a secondary electron (SE) detector. An atomic force microscope (AFM) from Veeco Metrology Group (Digital Instruments), operating in a tapping mode, was used for imaging the membranes topography. Differential scanning calorimetry (DSC) and thermal gravimetry (TG) were used to determine the thermal strength of the applied membranes (DSC-TG Simultaneous Testing Analyzer, STA 409C model from Netzsch). Membrane samples with ca. 0.6 mg weight were placed in corundum crucibles and heated in nitrogen atmosphere from 30 to 450 °C at the rate of 10 °C/min, according to the program set.

The degree of the membranes crystallinity was determined with the X-ray diffraction method, using DRON-2 X-Ray powder diffractometer. The measurements were performed for 2θ angles ranging from 5° do 60° at a 0.03° step record and the calculation time of 0.25 s, using a lamp with copper anode, fed with 25 mA and 45 kV current.

### 2.5. Calculations

Two models were applied for kinetic analysis of the metal ions transport through the membranes: Danesi model, based on Equation (1), and the model proposed by Szczepanski, based on Equation (2):

(1) $$\ln\frac{c_{t,f}}{c_{0}}=-\frac{P_{1}\cdot A}{V_{f}}t$$

(2) $$\ln\left(\frac{c_{t,f}-c_{e,f}}{c_{0}-c_{e,f}}\right)=-\frac{P_{2}\cdot A\cdot c_{0}}{V_{f}\left(c_{0}-c_{e,f}\right)}t$$

where c*_t,f_*—concentration of the metal ions in the feed phase at given time *t* (mol/m^3^); *c_0_*—initial concentration of the metal ions in the feed phase; c*_e,f_*—the equilibrium concentration of the metal ions in the feed phase (mol/m^3^); *P*—the permeability coefficient (m^2^/s); *A*—the membrane area (m^2^); *V_f_*—feed phase volume (m^3^).

The initial fluxes of the ions obtained during transport through PIM and SLM were calculated based on the following equation:

(3) $$J_{0}=P\cdot c_{0}$$

The extraction efficiency of the metal ion transport to the membrane phase (*E_m_*) and its re-extraction to the stripping phase (*RE_m_*) were calculated according to Equations (4) and (5), respectively:

(4) $$E_{m}=\frac{c_{0}-c_{t,f}}{c_{0}}\cdot 100\%$$

(5) $$RE_{m}=\frac{V_{s}\cdot c_{t,s}}{V_{f}\cdot c_{0}}\cdot 100\%$$

where c*_t,s_*—concentration of the metal ions in the stripping phase at given time *t* (mol/m^3^); *V_f_* —stripping phase volume (m^3^).

The selectivity of the membrane transport process was determined based on the value of the ion separation coefficient ($S_{m}$) expressed with the ratio of the initial fluxes of two transported ions of different metals—*M*_1_ and *M*_2_:

(6) $$S_{m}=\frac{J_{0,M_{1}}}{J_{0,M_{2}}}$$

The transport process activation energy was estimated based on the Eyring equation:

(7) $$\ln\;J_{0}=\frac{-E_{a}}{RT}+B$$

where *R*—gas constant (8.314 J/mol·K), *T*—temperature of the system (K); *B*—constant.

Diffusion resistance of the used membranes (Δ*_m_*) was calculated based on following Fick’s law form:

(8) $$\Delta_{m}=\frac{c_{c}\cdot A\cdot t}{V_{f}\left(c_{0}-c_{t,f}\right)}$$

where *c_c_*—concentration of the metal–ligand complex in the membrane (mol/m^3^).

The coefficient of the complex diffusion in the membrane (*D_m_)* was estimated as:

(9) $$D_{m}=\frac{d_{m}}{\Delta_{m}}$$

where *d_m_*—membrane thickness (m).

## 3. Results and discussion

### 3.1. Kinetics of Pb(II) Transport

**Figure 2:**
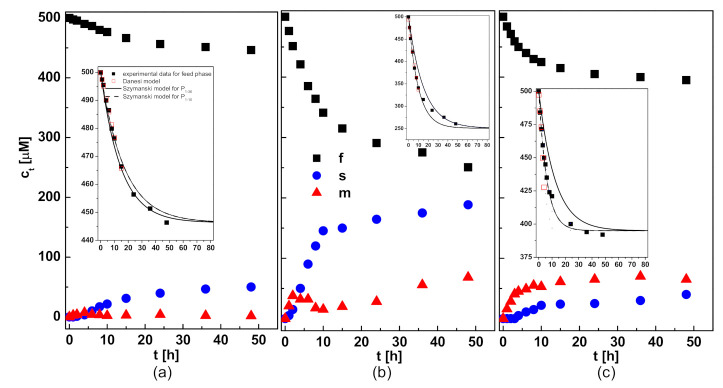
Changes in Pb(II) ion concentrations in each phase during transport through polymer inclusion membrane (PIM); feed phase (f): 500 µM solution of Pb(NO_3_)_2_, pH = 5.0; membrane phase (m): 2.0 cm^3^ o-NPOE/1 g CTA, 50 µmol/cm^3^ of (**a**) R7, (**b**) R7PS4 and (**c**) R7SP8; stripping phase (s): 0.50 M HNO_3._

Measurements were carried out of the ion concentration changes in the feed phase (*f*) and stripping phase (*s*), in the function of transport through PIM, in order to determine the kinetics of membrane release of Pb(II) ions. CTA-based membranes containing 2.0 cm^3^ (per 1 g CTA) *o*-nitrophenyl octyl ether (o-NPOE) as the plasticizer, and R7, R7PS4, R7PO4, R7PS8 and R7CEt8 carriers at 50 µmol per 1 cm^3^ of plasticizer were used. A 500 µM solution of Pb(II) ions with pH = 5.0 was the feed phase, while 0.50 M HNO_3_ was the stripping phase. The results of studies on Pb(II) ions re-extraction presented in [17] reveal a higher yield of their release from the metal solution with the pH = 5.0 to 1.0 M of HNO_3_ solution, but the use of the acid at the concentration exceeding 0.50 M as the stripping phase in transport through PIM caused its degradation. Sample changes in the Pb(II) ions concentration during transport through PIM with resorcin[4]arene carriers in the function of time are shown in Figure 2.

On the initial stage of Pb(II) ions transport through PIM containing functionalized resorcin[4]arenes, a significant decrease in the concentration of the tested ions concentration was observed in the feed phase, and the changes that occured in this period for all tested resorcinarenes were exponential in nature.

The usefulness of two kinetic models, proposed by Danesi [27] and Szczepanski [28], in the metal transport description was evaluated. According to the Danesi model, the ion transport can be described with a first-order kinetic reaction (Equation (1)) [27]. Model proposed by Szczepanski (Equation (2)) takes into account the volume of the feed phase and the membrane area and assumes reaching the equilibrium state of the system. Such an approach made it possible to estimate more accurately, compared to the Danesi model, the values of the initial fluxes of selected metal ions in transport through the PIM with a commercial carrier di-(2-ethylhexyl)phosphoric acid [28].

**Figure 3:**
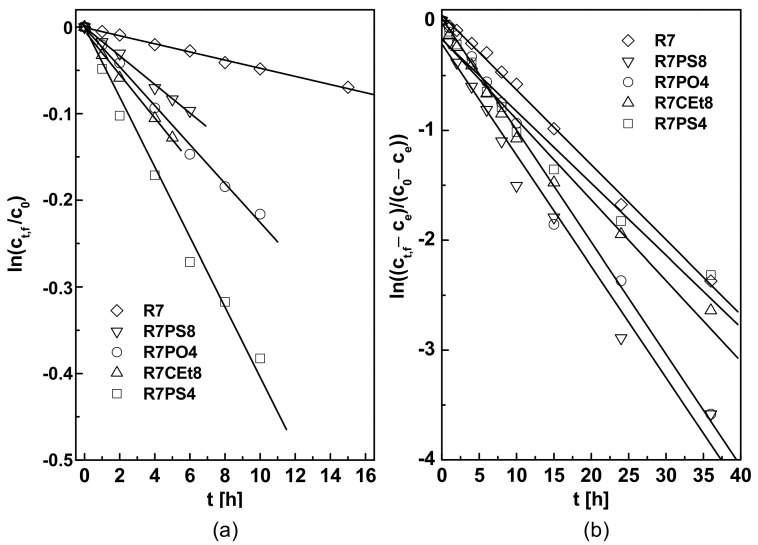
Logarithmic dependences of the metal concentration in feed phase on the time of Pb(II) ions transport through PIM with R7, R7PS4, R7PO4, R7PS8 and R7CEt8: (**a**) Danesi model, (**b**) Szczepanski model; transport conditions as in Figure 2.

The slopes of ln(*c_t,f_*/*c_0_*) = f(*t*) (Figure 3a) and ln ((*c_t,f_* − *c_0_*)/(*c_0_* − c_e_)) = f(*t*) (Figure 3b) relationships obtained for each applied membrane, in two independent experiments, were used to estimate the values of permeability coefficients (*P_1_* and *P_2,_* respectively) and initial fluxes of Pb(II) ions from the feed phase (*J_0_*), percent yield of the metal ions extraction to the membrane phase (*%E_m_*) and their re-extraction to the stripping phase (*%RE_m_*) were determined. The results of the calculations are summarized in Table 1.

The concentration of Pb(II) ions in the feed phase changed slightly during their transport through a membrane containing non-functionalized R7 resorcin[4]arene, and consequently, only 5% of Pb(II) ions were extracted to the membrane phase. The values of the initial fluxes of the tested Pb(II) ions through the membranes with R7PS4, R7PO4, R7PS8 and R7CEt8 carriers are higher than those obtained in the transport through PIM with the R7 carrier (Table 1), which is the evidence of a impact of resorcin[4]arene structure on the membrane extraction efficiency of the tested ions. The Danesi model is confirmed by a linear dependence of the natural logarithm from the ratio of the transported ion concentration in the feed phase to its initial concentration in the function of the transport time (Figure 3a), with the determination coefficients (R^2^) over 99.2% obtained for the initial 15 h of Pb(II) transport through PIM with R7, 10 h of Pb(II) transport through PIM with R7PS4 and R7PO4, 6 h of Pb(II) transport through PIM with R7PS8 and 5 h of Pb(II) transport through PIM with R7CEt8. Once the abovementioned transport time expires, the nature of the Pb(II) ions concentration changes suggests a zero-order reaction.

According to the Szczepanski model (Equation (2)), the permeability coefficients (P_2_) were estimated based on dependences of ln((*c_t,_f − c*0)/(*c*_0_ − *c*_e_)) vs. time for two ranges of time: 10 h (*P*_2–10_) and 36 h (*P*_2–36_). Better linearity of these relationships was obtained for the experimental data collected during the first 10 h of the transport through PIMs with functionalized resorcinarenes in comparison with the data obtained for 36 h of transport time (Table 1). In the case of the system with non-functionalized carrier R7, the Szczepanski model fitted the experimental data better when the *P*_2–36_ was taken into account (Figure 2a). For the membranes with R7PS4 and R7PO4, a good fit of the *c_t_* values calculated from Equation (2) and *P*_2–10_ to the experimental data was observed only in the first stage of the transport process (Figure 2b,c). Therefore, in the case of further experiments, the Danesi model was applied for calculations of the membrane permeability coefficient and the initial flux values.

**Figure 4:**
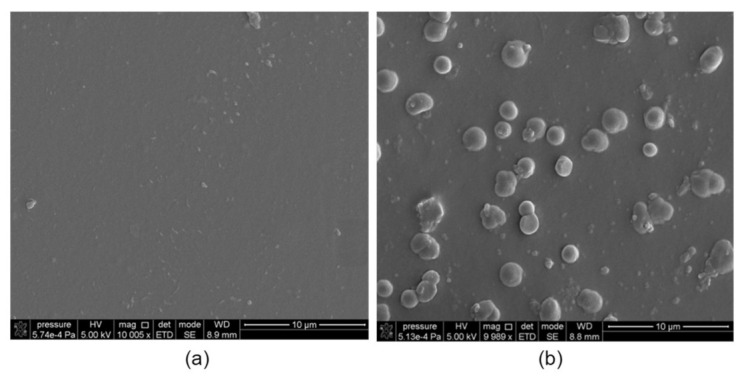
SEM images of the surface of polymer inclusion membranes containing (**a**) R7PS4 and (**b**) R7PS8, following a 48-h transport of Pb(II) ions.

**Figure 5:**
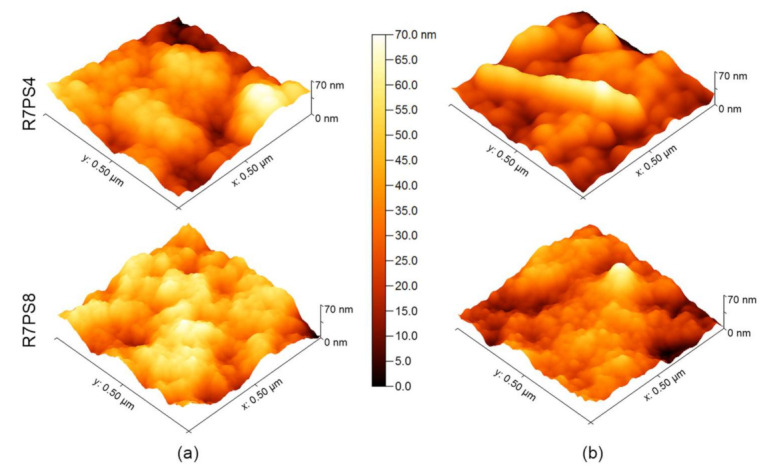
AFM images of polymer inclusion membranes containing R7PS4 and R7PS8 (**a**) before and (**b**) following a 48-h transport of Pb(II) ions.

The probable cause of the changes in the ions transport kinetics is a gradual saturation of the membrane organic phase with metal–resorcin[4]arene complexes, and final clogging of the membrane pores as a result of low efficiency of Pb(II) ions re-extraction from the membrane phase to the stripping phase (Table 1). Despite the fact that, after 10 h of transport, the pertraction of Pb(II) ions through PIM with R7PS4 and R7PO4 had an almost quantitative course, the re-extraction efficiency of the tested ions to the stripping phase as compared to their extraction to the membrane phase decreased, which caused an increase in the Pb(II) ions concentration in the membrane (Figure 2). Evident changes in the membrane appearance are the effect of Pb(II) ions accumulation in the membrane phase; it is particularly evident for the membranes with octasubstituted R7PS8 and R7CEt8 resorcinarenes. After a 48 h period of the tested ions transport through PIM with the abovementioned carriers, the initially transparent membranes turned opaque. The images of the membranes with R7PS4 and R7PS8 after Pb(II) ions transport, made with a scanning electron microscope (SEM) (Figure 4) and atomic force microscope (AFM) (Figure 5), clearly show the formation of deposits, which clog the membrane pores.

The folding degree of membranes with R7PS4 and R7PS8 (determined by *R_a_* parameter as the average absolute deviation of the roughness irregularities from the mean line over one sampling length parameter [29]) after the tested ions transport, amounted to 3.37 nm and 2.31 nm, respectively, and was lower than before the transport (3.64 nm and 4.09 nm, respectively), which is a testimony to gradual clogging of the membrane pores by the forming complexes of metal ions with the carrier.

### 3.2. Influence of the Stripping Phase Composition

In relation to the negligible efficiency of Pb(II) ions re-extraction from the membrane phase, containing octasubstituted carriers to 0.50 M of nitric acid solution as the stripping phase, an attempt was made to use other chemical compounds, which could enable a quick decomplexing reaction and transfer of the tested ions from the membrane phase to the stripping phase. The stripping phases such as ionized water, 0.50 M solutions of hydrochloric acid and sulfuric acid, 1.0 M of acetic acid and sodium acetate solutions and also 0.05 M ethylenediaminetetraacetate disodium salt (EDTA-Na) solution of pH = 3.0 (obtained by adding a small quantity of 1.0 M HNO_3_) were used. However, their application in the transport of Pb(II) ions from mono-ingredient solutions with 500 µM concentration and pH = 5.0 did not affect the efficiency of the process re-extraction. Only for the EDTA-Na solution as the stripping phase, after a 48 h transport through PIM with octafunctionalized R7PS8 and R7CEt8 resorcinarenes, a 4% increase in the Pb(II) ions re-extraction efficiency was observed, but the effect is not satisfactory enough to eliminate the problem of the membrane pores clogging by the formed complexes. That is why only the membranes containing tetrasubstituted resorcin[4]arenes R7PS4 and R7PO4 were selected for further studies on the transport process optimization.

### 3.3. Influence of the Membrane Phase Composition

The membrane phase composition (i.e., the type and quantity of the plasticizer and the metal ions carrier, as well as the type and thickness of the applied polymer support) is an important factor which affects the efficiency of metal ions removal from aqueous solutions during transport through polymer inclusion membranes.

#### 3.3.1. Plasticizer

**Figure 6:**
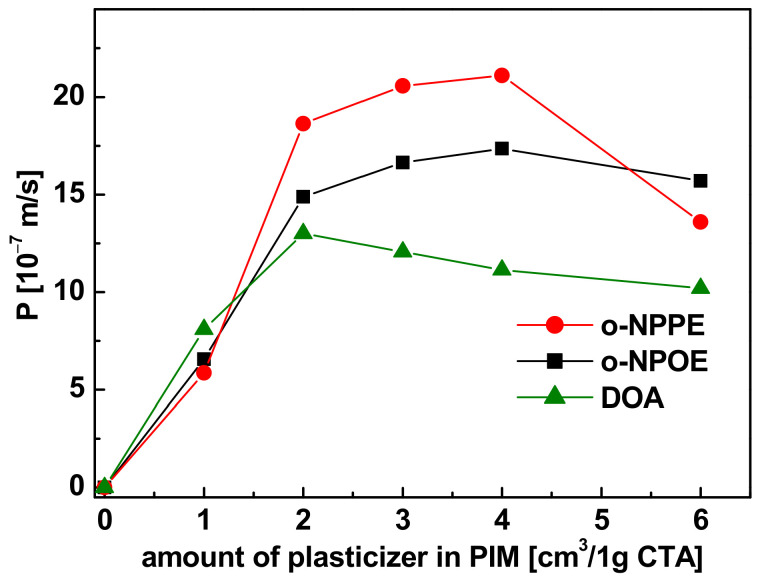
Influence of the plasticizer type and its quantity on the Pb(II) ions permeability through PIM; feed phase: 500 µM solution of Pb(NO_3_)_2_ (pH = 5.0); membrane phase: 25.0 mg CTA, 50 µmol/cm^3^ R7PS4; stripping phase: 0.50 M HNO_3._

The first stage included the examination of the plasticizer type and quantity in the PIMs on the transport of Pb(II) ions from the feed phase, which were mono-component solutions of the ions at the concentration of 500 µM and pH = 5.0 to 0.50 M HNO_3_ as the stripping phase. As plasticizers of CTA membranes with a constant content of R7PS4 carriers (50 µmol/cm^3^), *o*-nitrophenyl octyl ether (o-NPOE), *o*-nitrophenyl pentyl ether (o-NPPE) and bis(2-ethylohexyl) adipate (DOA) in the amount ranging from 0 to 6 cm^3^ per 1 g CTA, were applied. The influence of the plasticizer type and its quantity on the permeability of PIM with R7PS4 is shown in Figure 6.

Non-plasticized CTA membranes did not reveal Pb(II) ion transport. The permeability of PIM for the ions was increasing significantly with a higher content of the plasticizers in the membrane to 4 cm^3^/1 g CTA for o-NPOE and o-NPPE (76 and 77% wt., respectively), and to 2 cm^3^/1 g CTA (60% wt.) for DOA, and decreased afterward. The membrane with a phenylresorcin[4]arene carrier, applied by Zawierucha et al. [25], was characterized by maximum permeability for Pb(II) ions, at the 2 cm^3^/1 g CTA o-NPOE content, but with a 6 times higher concentration of the carrier. In order to achieve the maximum capacity of Pb(II), Zn(II) and Cd(II) ions transport through PIM with crown ethers as the carrier, Ulewicz et al. [30,31] used membranes containing 2.6 cm^3^/1 g CTA o-NPPE of the plasticizer. The noticeable decrease in the permeability of membranes with high plasticizer content may be caused by the physical and chemical changes in the membrane support, resulting in the plasticizer leakage from the membrane phase to the aqueous phases [6]. The o-NPPE > o-NPOE > DOA series obtained for the membranes containing over 2 cm^3^ of plasticizer/1 g CTA corresponds to the series obtained by Ulewicz et al. during Pb(II), Zn(II) and Cd(II) ions transport through PIM containing calix[4]-crown-6 [31]. The literature reports indicate an evident influence of the plasticizer viscosity and polarity on the efficiency of metal ions transport through PIM [6,23,32]. An increase in the plasticizer viscosity causes a decrease in the transport rate, most probably attributable to the slowdown in the ions diffusion in the membrane, whereas an increase in the plasticizers polarity has a positive impact on the metal ions decomposition inside the membrane, accelerating their transport rate. A lower value of the coefficient of viscosity (7.58 cP) and a higher dielectric constant (24) of o-NPPE [33] as compared to the other applied plasticizers (12.8 and 23.1 cP, respectively for o-NPOE [34], and 13.7 and 5.2 cP for DOA [33]) can make it a better ion carrier solvent and promote the formation of metal complexes in the organic membrane liquid phase. In relation to the above, plasticized o-NPPE membranes were used for further tests.

#### 3.3.2. Polymer Support

**Figure 7:**
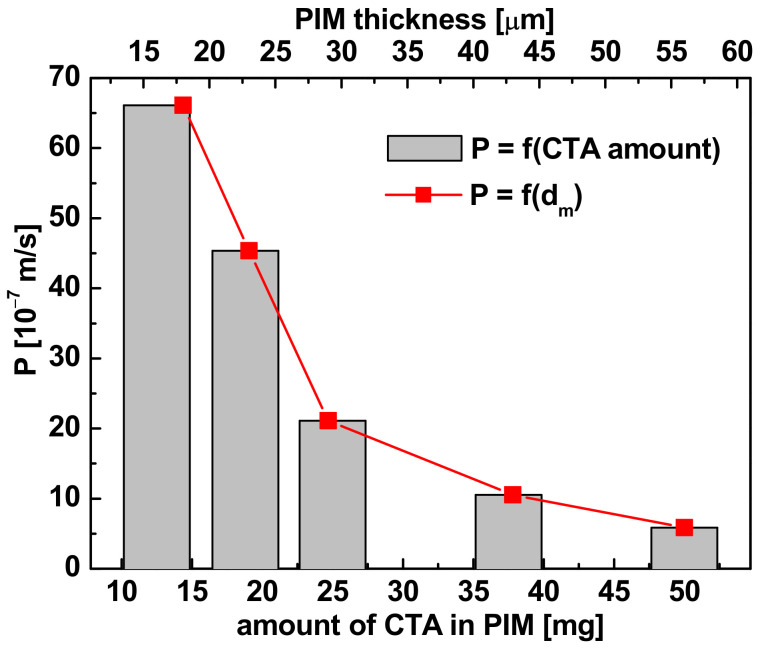
Influence of the CTA quantity in PIM and the membrane thickness on the transport of Pb(II) ions; feed phase: 500 µM solution Pb(NO_3_)_2_ (pH = 5.0); stripping phase: 0.50 M HNO_3_; membrane phase: CTA + 0.10 cm^3^ o-NPPE + 0.75 mg R7PS4.

Studies were carried out on the influence of quantity of CTA as the membrane support on the permeability of Pb(II) ions under transport conditions described in Section 3.3.1. PIMs containing a constant amount of the plasticizer (0.10 cm^3^ *o*-NPPE) and carrier (0.75 mg R7PS4), and a variable quantity of polymer (12.5–50.0 mg CTA). The observed changes in the permeability of the membranes depending on the CTA quantity and the membrane thickness are shown in Figure 7.

The permeability of the applied membranes, and consequently the value of the Pb(II) ions initial flux, decreased significantly with the polymer content in the membrane. The main cause of these changes is the increase in the membrane thickness with a simultaneous drop in the degree of the membrane plasticization. The highest values of the Pb(II) permeability were obtained for the membranes containing 12.5 mg and 18.8 mg CTA, but due to their low strength and stability upon contact with acidic aqueous phases, the membranes were not used in further tests. Consequently, polymer matrices prepared from 25 mg CTA were used in subsequent experiments.

#### 3.3.3. Carrier

**Figure 8:**
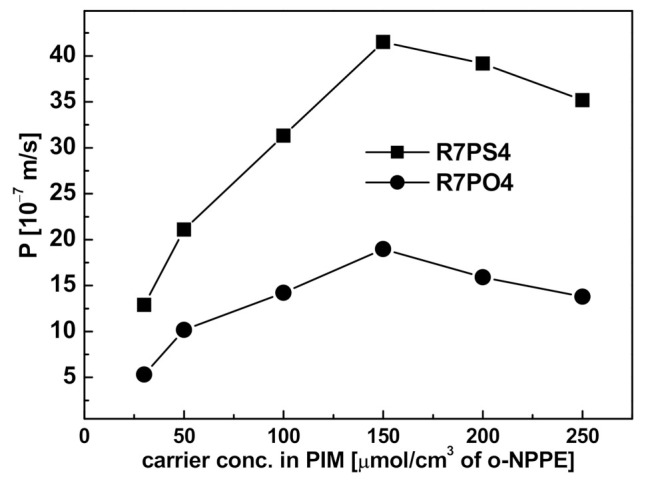
Influence of the carrier concentration on Pb(II) ions permeability through PIMs containing R7PS4 and R7PO4; feed phase: 500 µM solution of Pb(NO_3_)_2_ (pH = 5.0); membrane phase: 25 mg CTA + 4 cm^3^ o-NPPE/1 g CTA; stripping phase: 0.50 M HNO_3_.

The influence of the type and quantity of a resorcin[4]arene carrier in a polymer inclusion membrane with R7PS4 and R7PO4 on Pb(II) ions permeability was examined. Similarly to the previous experiments, a 500 µM solution of Pb(NO3)2 with pH = 5.0 was the feed phase, while a 0.50 M solution of HNO_3_ was the stripping phase. Membranes with a constant content of CTA (25 mg) and the plasticizer (4 cm^3^ *o*-NPPE/1 g CTA), and a variable concentration of the carrier in the membrane (from 0 to 250 µmol/cm^3^ of the plasticizer) were prepared. Each experiment was carried out for 6 h. The carrier-free membranes did not transport the Pb(II) ions, which confirms that the carrier concentration has a crucial influence on the metal ions transport through PIM. The permeability values of the studied ions increased with resorcin[4]arene concentration and reached the maximum values for the membranes containing 150 µmol/cm^3^ R7PS4 or R7PO4; however, the permeability of PIM with R7PS4 was twice higher for R7PS4 (4.15 and 1.90 µm/s, respectively for R7PS4 and R7PO4) (Figure 8).

A further increase in the carrier concentration in PIM caused a decrease in the parameters determining the Pb(II) ions transport rate. This phenomenon is described in the literature and explained by an increase in the membrane organic phase viscosity, and hence the membrane resistance reducing the complex diffusion through the membrane [35], or by a change in the transport mechanism from a diffusion to a step mechanism, caused by the carrier crystallization inside the membrane [36,37].

For Pb(II) ions transport through polymer inclusion membranes containing R7PS4 and R7PO4 carriers, where re-extraction of the transported metal ions from the membrane phase to the stripping phase occurs almost quantitatively, it is possible to estimate the diffusion resistance of the employed membranes (Δ*_m_)* and the coefficients of ion diffusion (*D_m_)* using Equations (8) and (9), respectively. The values of Δ*_m_* and *D_m_* obtained for Pb(II) ions transport through a 37 µm thick PIM with R7PS4 amount to 5.74 × 10^5^ s/m and 6.45 × 10^−11^ m^2^/s, respectively, and to 9.81 × 10^5^ s/m and 3.67 × 10^−11^ m^2^/s for a 36 µm thick PIM with R7PO4, revealing a significant influence of the diffusion rate of the resorcin[4]arene–metal ion complex through the membrane on the whole transport process rate.

**Figure 9:**
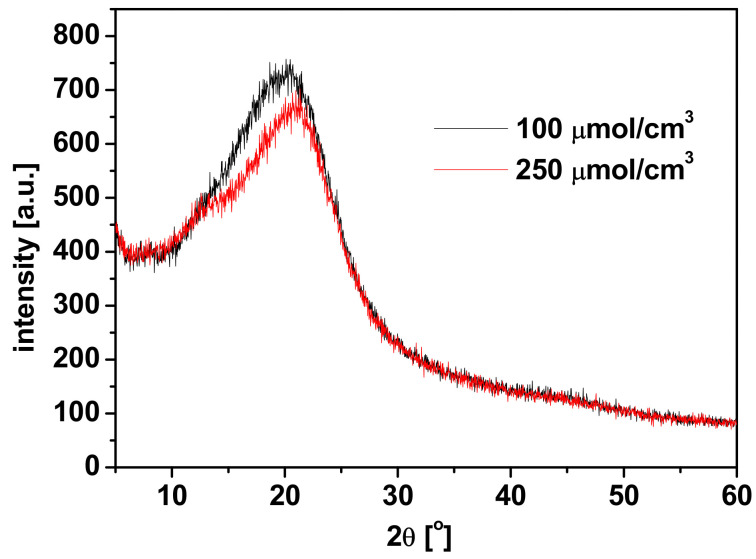
Diffractograms of PIMs with R7PS4 carrier at the concentration of 100 and 250 µmol/cm^3^.

The obtained values of the coefficients of diffusion are comparable with the literature data for Pb(II) ions transport through PIM with commercial TOPO [34], D2EHPA [38] and KELEX 100 [39] carriers and slightly lower than for the ions transport through PIM with *p*-*t*-butylcalix[4]arene [40]. The diffractogram of PIM with 100 µmol/cm^3^ R7PS4 shown in Figure 9 reveals the amorphous structure of the membrane, which eliminates the step mechanism of Pb(II) ions transport.

An increase in the carrier concentration in the membrane to 250 µmol/cm^3^ causes changes in the X-ray diffraction on the membranes, which can be the evidence of the presence of small quantities of crystalline forms in the membrane, promoting the step transport mechanism of Pb(II) ions. Hence, an increase in the carrier concentration leads to a change in the membrane morphology and thus affects the nature of the diffusion process. In relation to a lack of covalent bonds between the carrier and the polymer support, it can be assumed that the current mechanism of transport through the membrane is an intermediate mechanism between the diffusion of Pb(II)-resorcinarene complex and transition of Pb(II) ions between the resocinarene molecules [6].

The logarithmic dependence of the initial flux on the carrier concentration in the membrane enables the determination of the stoichiometry of the complexes formed during metal ions transport through the membrane. The relationships obtained for Pb(II) ions transport through the membranes containing less than 150 μmol/cm^3^ of the carriers were found to be linear with determination coefficients (R^2^) above 98.7%. Values of the straight-line slope (0.70 ± 0.05 and 0.79 ± 0.03, respectively, for R7PS4 and R7PO4) suggest a stoichiometry of the formed metal-resorcinarene 1:1 complexes.

For R7PO4, the stoichiometry of the 1:1 complex complies with the one obtained in the liquid–liquid extraction array (results not published), whereas the stoichiometry of Pb(II) ion complexes with R7PS4 varies from the one determined based on the solvent extraction results, where two R7PS4 molecules participated in the reaction of a single Pb(II) ion complexing [17]. It can be then concluded that the stoichiometry of Pb(II) complexes with the carrier depends on its concentration in the organic phase.

Taking into account the present studies and previous studies on solvent extraction of Pb(II) ions with resorcinarenes [17], indicating the capability of the tested macrocycles to co-transport NO_3_^−^ ions present in the feed phase, the reaction of Pb(II) ions complexing with resorcinarene ligands (L) on the feed phase/membrane border can be presented in the following general form:

[Pb^2+^]_aq_ + 2 [NO_3_^−^]_aq_ + [L]_org_ → [Pb(NO_3_)_2_L]_org_ (10)

### 3.4. Influence of the Metal Concentration in the Feed Phase

For PIM containing R7PS4 carriers, the dependence of Pb(II) ions release efficiency on their concentration in the feed phase was determined using 100, 250, 500 and 1000 µM solutions of Pb(NO_3_)_2_ with pH = 5.0 and 0.50 M solution of HNO_3_ as the stripping phase. An increase in Pb(II) ions concentration in the feed phase from 100 to 1000 µM caused a decrease in the Pb(II) extraction to the membrane phase from 94% to 38% and re-extraction of these ions to stripping phase from 89% to 35%. The nature of the changes in the values of the Pb(II) ions initial fluxes through PIM in the function of metal concentration in the feed phase (*c*_0_) in a logarithmic system is linear (R^2^ = 0.9950), with a slope equal to 0.75, suggesting that one Pb(II) cation participates in the reaction occurring on the feed phase/membrane border (a first-order reaction because of metal ions). This result confirms the stoichiometry of Equation (10) and the stoichiometry obtained in Pb(II) ions solvent extraction [17].

### 3.5. Influence of the Temperature

The process temperature is another factor that may have a crucial impact on the efficiency of metal ions transport through polymer inclusion membranes. In relation to the above, Pb(II) ions were transported through membranes with R7PS4 and R7PO4 carriers at five different temperature values, ranging from 25.0 to 50.0 °C. The initial fluxes of the tested ions increase as the temperature increases, to reach the maximum value at 50 °C, amounting to 5.71 and 3.5 µmol/m^2^s, respectively, for R7PS4 and R7PO4. The values of activation energy for the process (*E_a_*), estimated based on the slope of logarithmic dependence of Pb(II) ions initial fluxes on the temperature inverse (Equation (7)), amounting to 38.7 ± 1.3 and 36.1 ± 2.0 kJ/mol, respectively, for R7PS4 and R7PO4, suggest the transport processes are controllable by both diffusion and chemical reaction [41]. Similar values of activation energy were reported in the literature for alkali metals transport through SLM membranes containing calix[4]arene with four ester groups or calix[4]arenes with crown-5 [42]. A lower value of activation energy estimated at 19.1 kJ/mol obtained for transport through a PIM containing phenylresorcin[4]arene [25] reveals a diffusive nature of the process.

**Figure 10:**
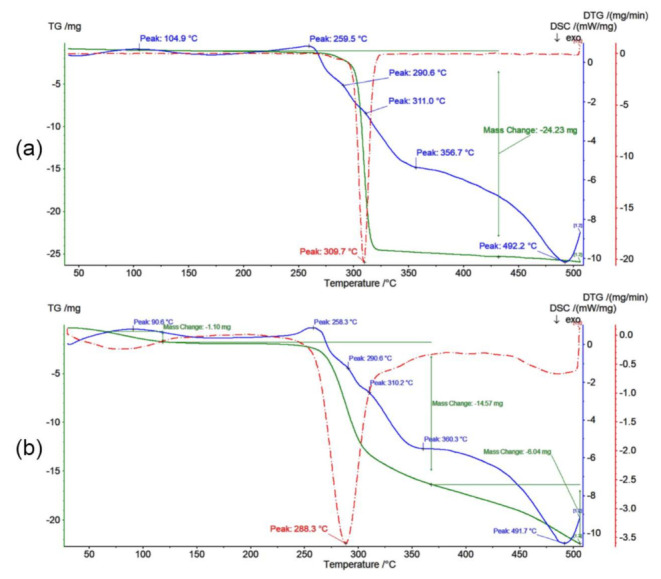
TG/DTG/DSC curves of polymer inclusion membranes with (**a**) R7PS4 and (**b**) R7PO4 carriers.

A thermal (DSC), thermal gravimetry (TG) and differential thermogravimetry (DTG) analyses were carried out on the applied PIMs to determine their thermal stability. DSC, TG and DTG curves of the membranes containing tetrafunctionalized resorcinarenes R7PS4 and R7PO4 are shown in Figure 10, whereas Table 2 summarizes the parameters that describe thermal characteristics of the test membranes.

Polymer inclusion membranes containing resorcin[4]arene carriers reveal a high thermal stability. Heating the PIM to 150 °C caused only a minor loss in its weight (maximum 3.80% for PIM with R7PO4), resulting most probably from evaporation of water left after the membrane conditioning. Thermal degradation of the tested PIMs occurs in several stages at temperatures over 250 °C and depends on the applied resorcinarene. The onset temperature of the tested PIMs instability, above which the highest weight loss was observed, is lower for a membrane with R7PO4 than for PIM with R7PS4, whereby the decomposition rate of the membrane with R7PS4, determined based on the TG curve inclination, is higher. Thermal decomposition of the tested membranes is an irreversible process, which occurs under the influence of heat supplied from the outside on the first stage, whereas on the subsequent stage, it takes place under the influence of heat released during combustion. According to the literature data, the main decomposition of non-plasticized CTA support occurs at 292–320 °C [26,36], whereas CTA membranes plasticized with *o*-NPPE reveal thermal stability only up to ca. 200 °C [43]. Therefore, it can be concluded that the introduction of resorcin[4]arene R7PS4 and R7PO4 into a membrane improves the thermal stability of the PIM.

### 3.6. Separation of Pb(II), Zn(II), Cd(II) and Cr(III) Ions

**Figure 11:**
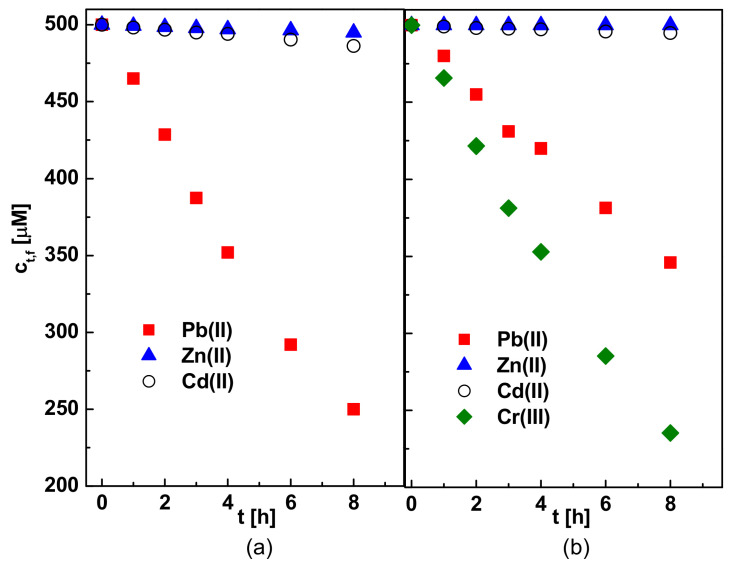
Metal ions separation from 500 µM solutions of (**a**) Pb(II), Zn(II) and Cd(II) and (**b**) Pb(II), Zn(II), Cd(II) and Cr(III); membrane phase: 25 mg CTA, 4 cm^3^ o-NPPE/1 g CTA, 150 µmol R7PS4/1 cm^3^ o-NPPE; stripping phase: 0.50 M HNO_3_.

Separation capabilities of R7PS4 resorcinarene were also investigated as the most effective carrier of Pb(II) ions in a competitive transport of metal ions from tri- and four-component feed phases with pH = 5.0, constituting a 500 µM nitrate solution of Pb(II), Zn(II) and Cd(II) or Pb(II), Zn(II), Cd(II) and Cr(III) to a 0.50 M solution of HNO_3_. A PIM containing a 150 µmol R7PS4/1 cm^3^ o-NPPE was used for this purpose. Results of these studies are presented in Figure 11 and Table 3.

The changes in the concentration of the transported metal ions in the feed phase, as a function of transport time through PIM with R7PS4 carrier, shown in Figure 11, reveal that the selectivity of Pb(II), Zn(II), Cd(II) and Cr(III) ions release depends primarily on the feed phase composition.

The presence of Cr(III) ions in the feed solution prevents a release and separation of the tested metal ions using PIM with R7PS4. The phenomenon could be caused by the formation of durable Cr(III)–R7PS4 complexes in the membrane phase, which are not subject to decomplexation on the stripping phase side and remain inside the membrane, blocking its pores. WD-XRF analysis of the membrane composition after metal ions transport from the feed phase containing Pb(II), Zn(II), Cd(II) and Cr(III) ions revealed 0.41% and 0.22% (by wt.) chromium and lead, respectively. R7PS4, characterized by the highest efficiency of Pb(II) ions membrane pertraction among the tested resorcinarenes, could be used for Pb(II), Zn(II) and Cd(II) ions separation on the condition that the feed solution does not contain Cr(III) ions.

### 3.7. Membrane Stability

The operation stability is an important factor which determines the possibility of commercial use of polymer inclusion membranes with resorcinarene carriers. Despite the fact that PIM membranes are considered to be more stable than SLM, in some metal ions, transport conditions washing out of the carrier was observed from the organic phase filling the PIM support pores, resulting in a decreased rate of the tested ions release [44]. This is why it seems purposeful to determine the stability of PIMs containing resorcinarene carriers, and to compare them with SLM type of membranes.

**Figure 12:**
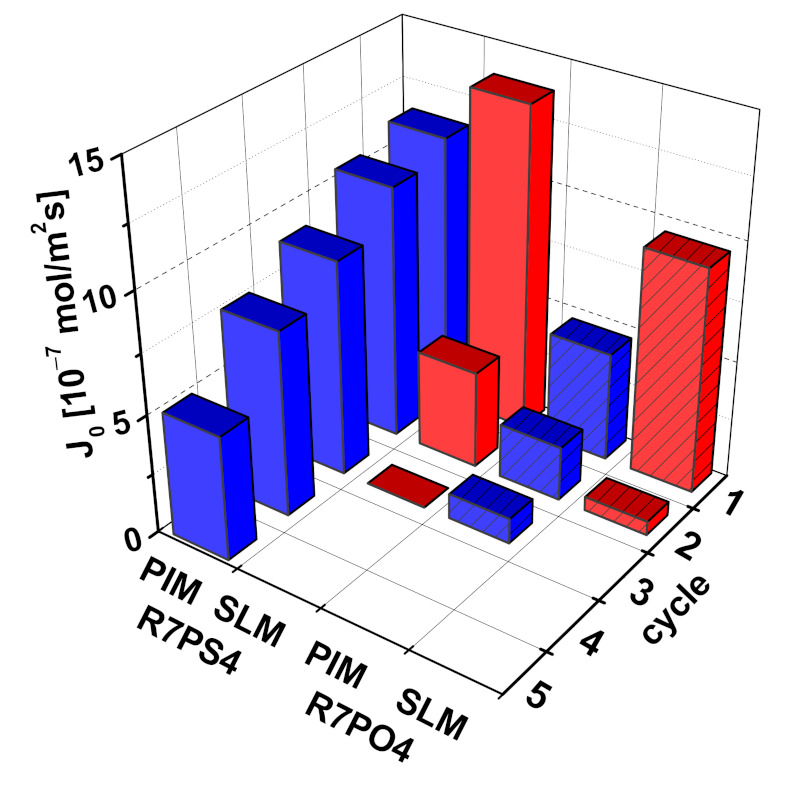
Changes in the values of Pb(II) ions initial fluxes in subsequent measurement cycles; feed phase: 500 µM Pb(II), Zn(II) and Cd(II) solution, pH = 5.0; stripping phase: 0.5 M HNO_3_, membrane: SLM—0.10 M R7PS4 or R7PO4 solution in trichloromethane, PIM—22.5 g CTA, 1.56 cm^3^ o-NPPE/1 g CTA, 100 µM R7PS4/cm^3^ o-NPPE.

In order to determine the stability of PIMs containing resorcinarene carriers, tests were carried out on a competitive transport of Pb(II), Zn(II) and Cd(II) ions in five measurement cycles (10 h each), using the same membrane containing 35% CTA, 56% o-NPPE and 9% of R7PS4 carrier in subsequent cycles. Such a PIM composition helped to obtain membranes with parameters comparable with SLM made of Celgard 2500 sheet (PIM: thickness: 27 µm, porosity estimated based on the organic phase volume share in the whole membrane volume: 56%; SLM: thickness: 25 µm, porosity: 55%). Before each cycle of the process, the PIM membrane was conditioned in distilled water, and the aqueous phases were replaced with new ones. The values of Pb(II) ion transport fluxes through PIM and SLM, determined in subsequent measurements, from equimolar (500 µM) Pb(II), Zn(II) and Cd(II) solutions with pH = 5.0 to 0.50 M HNO_3_ are shown in Figure 12.

Despite the fact that the initial flux of Pb(II) ions in the first measurement cycle was higher for SLM membranes than for PIMs, it dropped by 70% in the following cycle, which basically excluded further usability of the membranes. The instability of SLM membranes containing resorcinarene carriers can be caused, similarly to other cases of membranes described in the literature, by a loss of the solvent or carrier from the membrane phase, as a result of the difference in the osmotic pressure on both sides of the membrane, or a solution leakage as a result of water channels formation between the feed phase and the stripping phase [45,46]. The obtained values of Pb(II) ions initial fluxes in the transport through PIM membranes were gradually decreasing with each subsequent work cycle, from the value of 1.14 µmol/m^2^s in the first cycle to 0.52 µmol/m^2^s in the last cycle.

Polymer inclusion membranes containing non-functionalized R7 resorcinarene used by Benosmane et al. [23] in the transport of Pb(II) ions revealed stability for 120 h, followed by a 20% decrease in the initial flux of the tested ions. The membrane containing phenylresorcin[4]arene carrier used in Pb(II) ions transport from one-component feed phase was characterized by stable operation for 90 h [25]. The stability of PIM and SLM membranes containing macrocyclic carriers are rarely compared in the literature. PIM type membranes with crown ethers as ion carriers, used by Arous et al. [36] to transport Ag(I), revealed better stability (over 2 weeks) as compared to SLM membranes, for which the initial flux of the tested ions dropped after 4 days. Kim et al. [47], who released Cs(I) ions through PIM and SLM with calix[4]-crown-6, demonstrated the working time of PIM to be much longer (15 days) than that of SLM (1 day).

## 4. Conclusions

The presented studies helped to assess the fitness of the selected resorcin[4]arenes for efficient and selective Pb(II) removal from diluted aqueous solutions during transport through polymer inclusion membranes.

The obtained study results indicate that the efficiency of the tested ions transport by means of non-functionalized resorcin[4]arenes with heptyl chains is lower than for the derivatives functionalized with phosphoryl, triphosphoryl or ester groups, whereby tetrathiophosphorylated resorcin[4]arene R7PS4 is the most efficient carrier of Pb(II) ions. Moreover, the efficiency of Pb(II) ions release from aqueous systems clearly depends on the composition of the feed, membrane and stripping phase and on the system temperature. The determined activation energy values suggest a diffusion-reactive mechanism of Pb(II) ions transport through PIM with tetrasubstituted R7PS4 and R7PO4 resorcinarenes. It was also discovered that the applied membranes are characterized by thermal stability up to the temperature of ca. 250 °C, and the working time of PIM containing R7PS4 resorcinarene at a room temperature is lower than the life of SLM-type membranes that contain the same carrier. Despite the fact that the coefficients of Pb(II) and Cr(III) ions transport selectivity towards Zn(II) and Cd(II), estimated based on the values of the metal ions initial fluxes, suggest the possibility of the ions separation, the formation of durable resorcinarene complexes with Cr(III) ions, causing clogging of the membrane pores, significantly reduces the efficiency of re-extraction to the stripping phase, eliminating the fitness of the applied PIMs for selective release of Pb(II), Zn(II) and Cd(II) ions from solutions containing Cr(III) ions.

The obtained results create a new perspective for further work on polymer inclusion membranes containing resorcin[4]arenes as carriers of heavy metal ions. The continuation of the presented studies, especially in terms of improving the efficiency of metal ions extraction into the receiving phase and the membrane stability, is of key importance in the perspective of the possibility of using the tested systems on a scale larger than the laboratory, e.g., in water and wastewater treatment plants.

## Figures and Tables

**Table 1:** Parameters of Pb(II) ions transport through PIM with calixresorcin[4]arene carriers.

Parameter\ Carrier	R7	R7PS4	R7PO4	R7PS8	R7CEt8
*E_m_* [%]	5	32	21	14	23
*RE_m_* [%]	4	30	20	5	7
Danesi model					
*t* [h]	15	10	10	6	5
*P*_1_ [10^−7^ m/s]	1.70	14.89	8.33	6.22	9.33
*J*_0_ [10^−7^ mol/m^2^s]	0.85	7.44	4.17	3.11	4.67
*R^2^* [%]	99.57	99.29	99.37	99.52	99.39
Szczepanski model for *t* = 36 h					
*P*_2–36_ [10^−7^ m/s]	2.71	12.13	9.52	3.64	6.62
*J*_0_ [10^−7^ mol/m^2^s]	1.36	6.06	4.76	1.82	3.31
*R*^2^ [%]	99.62	96.29	97.86	95.06	96.96
Szczepanski model for *t* = 10 h					
*P*_2–10_ [10^−7^ m/s]	2.32	18.55	9.36	6.38	8.50
*J*_0_ [10^−7^ mol/m^2^s]	1.16	9.28	4.68	3.19	4.25
*R*^2^ [%]	99.02	99.84	99.64	97.29	97.90

**Table 2:** Thermal characteristics of polymer inclusion membranes with calixresorcin[4]arenes.

Carrier	TG			DTG	DSC	
	Temperature Range [°C]	Weight Loss [%]	Decomposition Rate Constant (tgα)	dm/dt [mg/min]	Temperature [°C]	∆H [J/g]
R7PS4	25–150	0	0	-	104.9	+18.6
	150–290	0	0	-	259.5 290.6	+25.9 +1.8
	290–350	76.3	4.64	24.23	311.0	+1.1
	350–500	0	0	-	356.7 492.2	−24.7 −78.4
R7PO4	25–150	3.80	0.11	1.10	90.6	+14.8
	150–260	0	0	-	258.3	+21.7
	260–350	50.4	3.84	14.57	290.6 310.2	+1.8 +1.6
	350–500	20.9	0.71	6.04	360.3 491.7	−2.4 −74.5

**Table 3:** Initial fluxes and selectivity coefficients obtained in a competitive transport of Pb(II), Zn(II), Cd(II) and Cr(III) ions; process conditions as shown in Figure 11 (* a lack of metal ions re-extraction to the stripping phase).

System	Pb(II)	Zn(II)	Cd(II)	Cr(III)	*S_m_*	
	*J*_0_ [10^−7^ mol/m^2^s]					
Pb(II), Zn(II), Cd(II)	16.48	0.22	0.63	-	Pb(II)/Zn(II)	74.91
					Pb(II)/Cd(II)	26.15
Pb(II), Zn(II), Cd(II), Cr(III)	8.46	0	0.22	17.69	Cr(III)/Pb(II)	2.09 *
					Cr(III)/Zn(II)	∞ *
					Cr(III)/Cd(II)	80.41 *

## Data Availability

Data is contained within the article.
